# IRE made easy: introducing the robotic grid system for multiple parallel needle insertion in irreversible electroporation treatment

**DOI:** 10.1007/s11548-024-03216-w

**Published:** 2024-06-19

**Authors:** Girindra Wardhana, Jurgen J. Fütterer, Momen Abayazid

**Affiliations:** 1 Robotics and Mechatronics Lab (RAM), TechMed Centre, University of Twente, Hallenweg 15, Enschede, 7500 NH Overijssel The Netherlands; 2 Department of Medical Imaging, Radboud University Medical Center, Geert Grooteplein Zuid 10, 6525 GA Nijmegen, Gelderland The Netherlands

**Keywords:** Irreversible electroporation, Robot-assisted intervention, Medical robotics, Percutaneous procedure

## Abstract

**Purpose:**

Accurate needle placement is crucial for successful tumor treatment using the irreversible electroporation (IRE) method. Multiple needles are inserted around the tumor, ideally in parallel, to achieve uniform electric field distribution. This paper presents a robot utilizing a grid system to enable multiple needles insertion while maintaining parallelism between them.

**Methods:**

The robotic system has two degrees of freedom, which allow for the adjustment of the grid system to accommodate targeting lesions in various positions. The robot’s performance was evaluated by testing its accuracy across various configurations and target depth locations, as well as its ability to maintain the needle parallelism.

**Results:**

The robot has dimensions of ${\phi }$ 134 mm and a height of 46 mm, with a total weight of 295 g. The system accuracy test showed that the robot can precisely target points across different target depths and needle orientations, with an average error of $2.71\pm 0.68$ mm. Moreover, multiple insertions at different grid locations reveal needle orientation deviations typically below $1^{\circ }$.

**Conclusion:**

This study presented the design and validation of a robotic grid system. The robot is capable of maintaining insertion accuracy and needle parallelism during multiple needle insertions at various robot configurations. The robot showed promising results with limited needle deviation, making it suitable for IRE procedures.

## Introduction

Irreversible electroporation (IRE) is an emerging soft tissue tumor treatment technique. In contrary to other heat-based ablation techniques, IRE applies short high-voltage electric pulses to destroy the tumor cell [1]. Multiple needles are placed in the surrounding of the tumor and an electric field is applied between the pair of needles. Typically, the number of needles varies from 2 to 6 depending on the tumor’s size and shape.

Precise needle positioning is a crucial factor in determining the efficacy of tumor ablation procedures. In IRE, needles should be placed parallel to each other to obtain homogeneous electric field distribution [2, 3]. Converge placement of the needle will increase the risk of over-current which will lead to temperature rise and may cause thermal damage to surrounding tissue. Meanwhile, diverging needle position will cause insufficient coverage of electric field which increases the risk of incomplete ablation.

In current medical practice, needle placement procedures are typically performed manually by clinicians. However, the utilization of robotic devices to aid in these procedures has been gaining popularity, as they have the potential to enhance accuracy and reduce the duration of the procedure. The integration of robotic devices with image guidance has been explored in several studies, such as in the case of cryoablation [4], shoulder arthrography procedures [5], and low back pain injections [6]. With regards to IRE, various solutions have been proposed, ranging from simple devices such as needle spacers to maintain the distance and orientation between needles, to more advanced approaches like navigation systems and robotic devices. Commercially available systems such as stereotactic computed tomography (CT)-guidance (CAS-ONE, CAScination AG, Switzerland) [7] and CT-guided robotic system (Maxio, Perfint Healthcare, USA) [8] have also been reported to provide accurate needle placement. These studies showed that robotic assistance results in a shorter procedure time, fewer punctures to the patient, and lower radiation dose compared to the manual placement of IRE probes.

Despite the recent advancement in needle placement technology, there is limited literature available on devices that support multiple needle insertions. For instance, small patient-mounted robots developed by He et al. [9] were used to perform multiple needle insertions for tumor treatment. They used several robots to insert multiple needles in various locations. This approach is not applicable for IRE due to the limitation of the distance between the needle pair, which range needs to be 10–25 mm. For the application of commercial systems in IRE, they performed needle placement by inserting the needle one by one. This approach requires proper adjustment to each needle, especially to maintain proper parallelism, in addition to accurate placement of the needle tip.

In response to this gap, this study proposes the development of a robotic grid system as a novel solution for multiple parallel needle insertion in IRE treatments. The proposed system has two degrees of freedom (DOF) implemented in the grid system to support the angulation of the needle path to accommodate insertion in complex multi-angle situations. This system represents an improvement over the previous robot that our research group has developed [10], where multiple needle insertion is previously achieved by inserting the needle one by one. With the current design, multiple needles can now be inserted simultaneously while maintaining parallelism between the needles by using a single device.

**Fig. 1:**
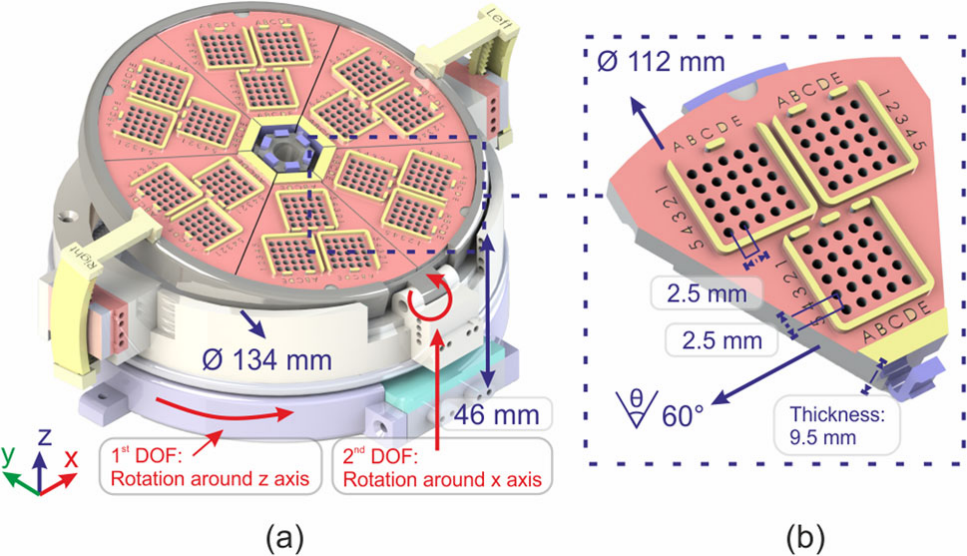
**a** Robotic grid system CAD model showing the degrees of freedom and the robot dimensions. **b** Subgrid element that has 3 main groups of $5\times 5$ array of holes with diameter of 1.5 mm

## Methodology

### Mechanical design and fabrication

The robot design was inspired by the precision grid used in breast biopsy. Figure 1 shows the design of the robot grid system. The precision grid provides a visual guide for the needle’s path, helping to ensure that the needle is placed in the correct location. Localized needle entry help to obtain a reproducible and consistent placement with the needle. In addition, the precision grid can be easily incorporated into the standard medical procedure and do not require special training to use. Furthermore, the main advantage of utilizing precision grid in the case of IRE is the capability of handling insertion of multiple needles at the same time with the same orientation, without the need for individual needle adjustment, which will reduce the number of reiterations and thus the procedure time. This was the main reason of choosing this concept to be implemented in our robot design.

In conventional precision grids, needle must follow the pre-determined path outline by the grid. It limits the maneuverability of the needle, especially when deviation occurred which reduce the accuracy of the needle placement. Moriera et al. [11] introduce an angulated needle-guide template for answering this problem, where the needle orientation can be adjusted by angulated grid template system instead of adjusted manually by the clinician. Although that approach can reduce the deviation of the needle, it supports only for a single needle insertion since the application is for prostate biopsy. We adopt the idea and improved the robot design in order to manage multiple needle insertion required in IRE procedure.

In our robot design, we incorporated pneumatic stepper motors to enable orienting the needle grid. These motors are using double acting cylinder mechanism, as presented in the work of Groenhuis et al. [12]. Each motor is controlled by four pneumatic tubes made of polyurethane. The robot system has two degrees of freedom (DOF), which consist of rotation around the *z*-axis (1st DOF) and *x*-axis (2nd DOF) as shown in Fig. 1a. The resolution of the motors is affected by the size of the stepper teeth or rack. In this design, the resolution for both stepper motor is 0.5^∘^, with a motor range of up to 360^∘^ for rotation in the *z*-axis and 20^∘^ for rotation in the *x*-axis.

The robot incorporates a grid system that comprises of six subgrids that are assembled together on the main robot frame. The subgrids have a diameter of 112 mm and thickness of 9.5 mm. They are attached to the robot frame using snap-fit mechanism, which allows for easy removal of unused subgrids to increase the available space in the robot’s workspace. It also allows for the subgrid to be sterilized independently without the need to include the whole system.

Additionally, this design facilitates individual modification to the size of the grid hole. Each subgrid is composed of three main groups of $5\times 5$ arrays of equally spaced holes with a distance of 2.5 mm. The current grid system has a hole diameter of 1.5 mm, to accommodate the common size of electrode diameter used in IRE (18G). A naming convention is used to identify the location of each hole within the subgrid, making it easy to locate and select the desired hole. For instance, a hole with ID of ‘5-iii-B4’ means the hole is located at subgrid 5, group iii, column B, and row 4.

The robot body, including the grid system and frame, were fabricated using a 3D printer with Makerpoint Ultimaker Tough PLA material (Makerpoint Holding, Wageningen, The Netherlands). The pneumatic motors and the racks were printed using Stratasys Objet Eden 260 with FullCure720 material (Stratasys Ltd, Eden Prairie, MN, USA). The components were assembled using nylon screws and bolts, resulting in a robot with a dimension of 134 mm in diameter and 46 mm in height, with a total weight of 295 g.

The robot is designed to assist in IRE treatment of abdominal tumors, including those in the liver and pancreas. Thanks to its lightweight design and MRI-compatibility, the robot can be directly mounted on the patient’s abdomen. Belts connected from the table to the strap slots in the robot’s base are used to secure the robot to the patient’s body.

### Kinematic analysis

A transformation matrix can be derived to calculate the position of the needle tip with respect to the robot’s coordinate system. Two variables, $q_{z}$ and $q_{x}$ represent the orientation of the robot frame in the *z* and *x*-axis, respectively. Other variables, such as $h_\text {axis}$ and $h_{y}$ are obtained from physical dimensions of robot body and are equal to 37.8 mm and 61.5 mm. $h_\text {axis}$ is the height of the x-axis rotation from the robot body, and $h_{y}$ is the shifting distance of the x-rotation from the center of the robot. By combining the transformation for each degree of freedom with the location of the selected grid hole and the length of the insertion, the following transformation matrix for the end effector can be obtained.

1 $$\begin{aligned}{} & {} \varvec{T}_{ee} = \varvec{T}_{q_{z}} \, \varvec{T}_{h_\text {axis}} \,\varvec{T}_{q_\text {x}} \, \varvec{T}_{q_\text {insert}}, \nonumber \\{} & {} \varvec{T}_{ee} = \begin{bmatrix} \varvec{R}_{q_{z}} &{} \quad \varvec{0}_{3\times 1} \\ \varvec{0}_{1\times 3} &{} \quad 1 \end{bmatrix} \begin{bmatrix} \varvec{I}_{3\times 3} &{} \begin{bmatrix} 0 \\ 0 \\ h_\text {axis} \end{bmatrix} \\ \varvec{0}_{1\times 3} &{} 1 \end{bmatrix}\nonumber \\{} & {} \begin{bmatrix} \varvec{R}_{q_{x}} &{} \left( \begin{bmatrix} 0 \\ h_{y} \\ 0 \end{bmatrix} - \varvec{R}_{q_{x}} \begin{bmatrix} 0 \\ h_{y} \\ 0 \end{bmatrix}\right) \\ \varvec{0}_{1\times 3} &{} \quad 1 \end{bmatrix} \begin{bmatrix} \varvec{I}_{3\times 3} &{} \begin{bmatrix} x_\text {grid} \\ y_\text {grid} \\ q_\text {insert} \end{bmatrix} \\ \varvec{0}_{1\times 3} &{} \quad 1 \end{bmatrix}, \end{aligned}$$

where $\varvec{I}$ is a $3\times 3$ identity matrix, $x_\text {grid}$ and $y_\text {grid}$ are the coordinate of the grid hole, and $q_\text {insert}$ is the length of needle insertion.

Rotation matrices are given by:

$$\begin{aligned} \varvec{R}_{q_\text {z}} = \begin{bmatrix} \cos {q_{z}} &{} \quad -\sin {q_{z}} &{} \quad 0\\ \sin {q_{z}} &{} \quad \cos {q_{z}} &{} \quad 0\\ 0 &{} \quad 0 &{} \quad 1\\ \end{bmatrix}, \varvec{R}_{q_{x}} = \begin{bmatrix} 1 &{} \quad 0 &{} \quad 0\\ 0 &{} \quad \cos {q_{x}} &{} \quad -\sin {q_{x}}\\ 0 &{} \quad \sin {q_{x}} &{} \quad \cos {q_{x}}\\ \end{bmatrix}. \end{aligned}$$

**Fig. 2:**
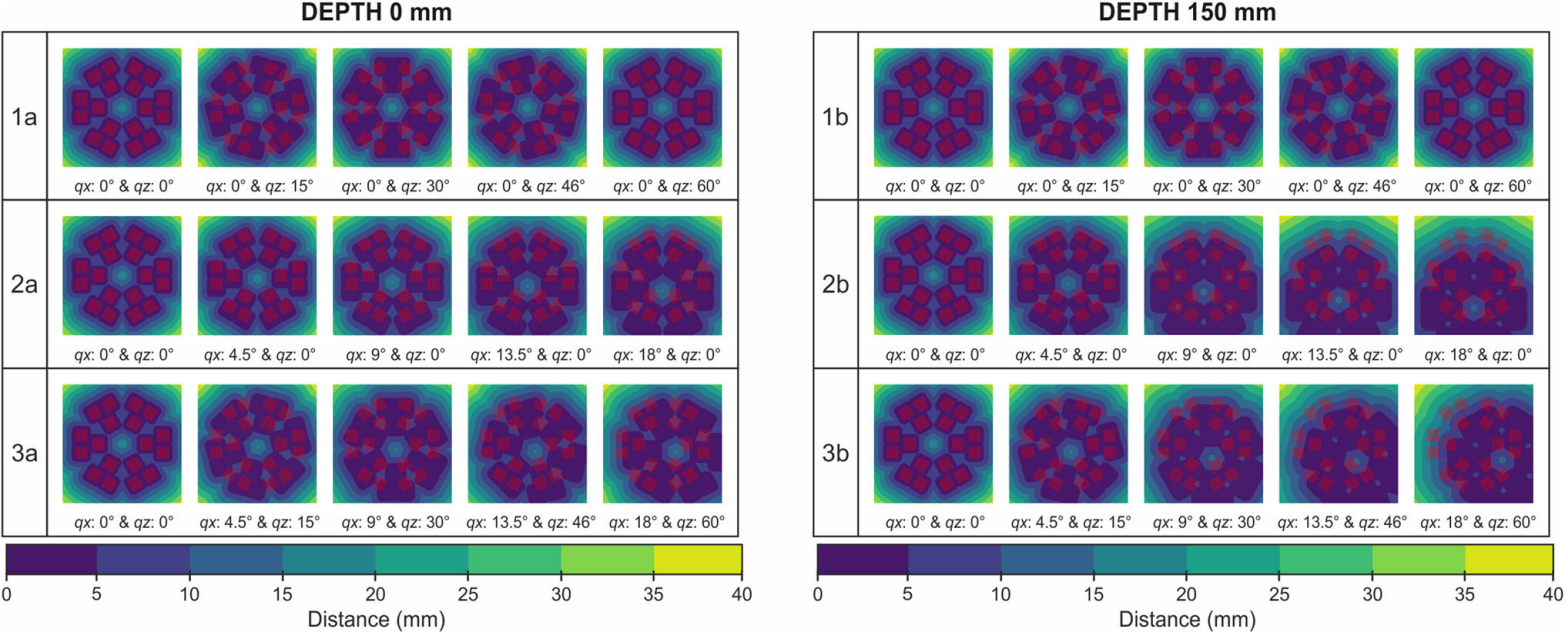
The reachable region of the robot grid at depths of 0 mm and 150 mm in various robot orientations, including (1a-1b) rotation only in the *z*-axis, (2a-2b) rotation only in the x-axis, and (3a-3b) combination of rotation in the *z* and *x*-axis. A group of red dot represents the position of the subgrids when no orientation is given to the robot

### Reachability analysis

The arrangement of the grid hole imposes limitations on the reachable regions under the robot body. However, this reachable region can be modified by adjusting the position of the grid system. By utilizing the derived transformation matrix for the end effector, it is possible to estimate this region in relation to the orientation of the grid.

The reachable region is determined through the mapping of distances between the needle tip and the surrounding area. This mapping method allows for the classification of specific locations as reachable or not reachable. In this study, three schemes were used to observe the reachable region with a particular emphasis on the area beneath the robot frame ($z=0$ mm) and the region typically affected by lesions ($z=150$ mm) as shown in Fig. 2. These schemes included rotating the robot grid:

1. only in the *z*-axis, from $0^\circ$ to $60^\circ$ with a $15^\circ$ increment
2. only in the *x*-axis, from $0^\circ$ to $18^\circ$ with a $4.5^\circ$ increment
3. combination of the *z* and *x*-axes using the values from scheme (1) and (2)

The reachable region from the robot grid varies with different grid orientations, as shown in Fig. 2. As illustrated from Fig. 2(1a,1b), the reachable region remains consistent for every 60^∘^, when rotation is applied only in the z-direction. The reachable region from $q_{z}=60^{\circ }$ yielding the same results as $q_{z}=0^{\circ }$, due to the grid design’s symmetry. On the other hand, applying rotation only in the *x*-axis, as depicted in Fig. 2(2a,2b), the reachable region shifts away from the x-axis, with the distance increasing with higher rotation. For difficult positions, such as areas located farther from the center of the grid, combining rotations in both the *z* and *x*-axes can provide an effective solution, as demonstrated in Fig. 2(3a,3b).

From this result, it is worth to mention that the rotation in the *x* axis has an important role on reaching region that located further away form the robot center. Also, the result is mainly correlated by the depth of the target. The result of the second and third scheme show significant differences between the targets located underneath the robot frame and those in deeper locations. Furthermore, it is important to note that reaching a position in the corner by adjusting the orientation of the grid in *x* and *z*, will give a unique pose to the needle position and orientation. If the target required specific path to be followed, it will be easier to readjust the robot system and redo the planning.

## Experiments

Two sets of tests were performed to evaluate the robot grid performance: a system accuracy test and a needle alignment test. The former analyzed the capability of the robot to precisely target designated points. The latter examined the ability of the robot to maintain needle parallelism, a vital aspect for the successful completion of IRE treatment. For both tests, various configurations of the robot and depths of the targets were considered to evaluate the robot’s performance.

### Experimental setup

**Fig. 3:**
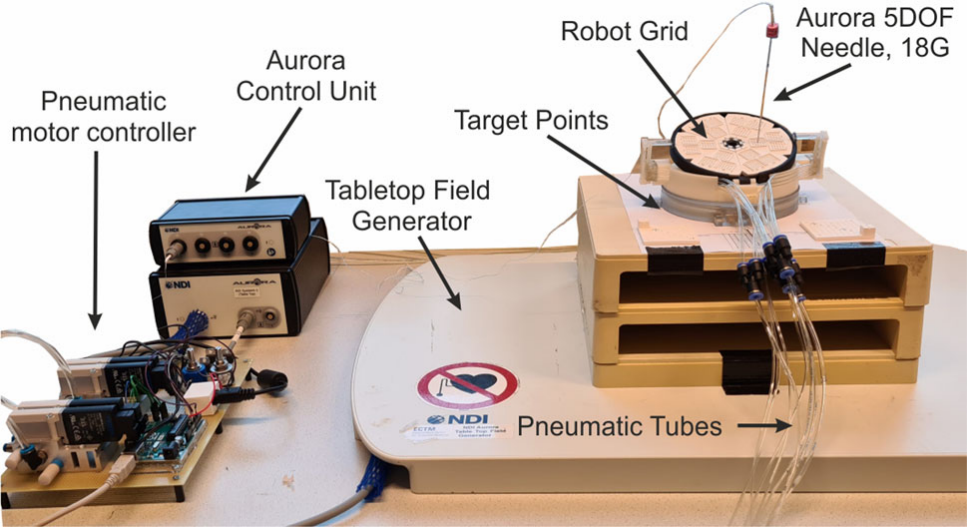
Experiment setup for evaluating the robot grid system, including robotic system, Aurora electromagnetic tracker system, and control board for pneumatic motors

Figure 3 shows experimental setup for robot grid evaluation. The robot grid is operated through the Robot Operating System (ROS) Melodic. The robot setup is actuated with 3D printed pneumatic motor using air pressure of 3 bar. The activation of the pneumatic valve was controlled by an Arduino Mega 2560, which was connected to ROS.

The robot body was located on the Tabletop Field Generator, which is an electromagnetic (EM) tracking system as part of Northern Digital’s Aurora (NDI, Waterloo, Canada). The position and orientation of the needle tip were tracked using the Aurora 5-DOF needle. With a length of 15 cm, the needle sensor corresponds to the typical electrode length used in the NanoKnife system for IRE treatment. Additionally, according to Xu et al. [13], the mean distance from skin to liver lesion in patients undergoing treatment for liver metastases was 10.7 cm (7.4–14.6 cm), which is within the reach of this needle’s length.

**Fig. 4:**
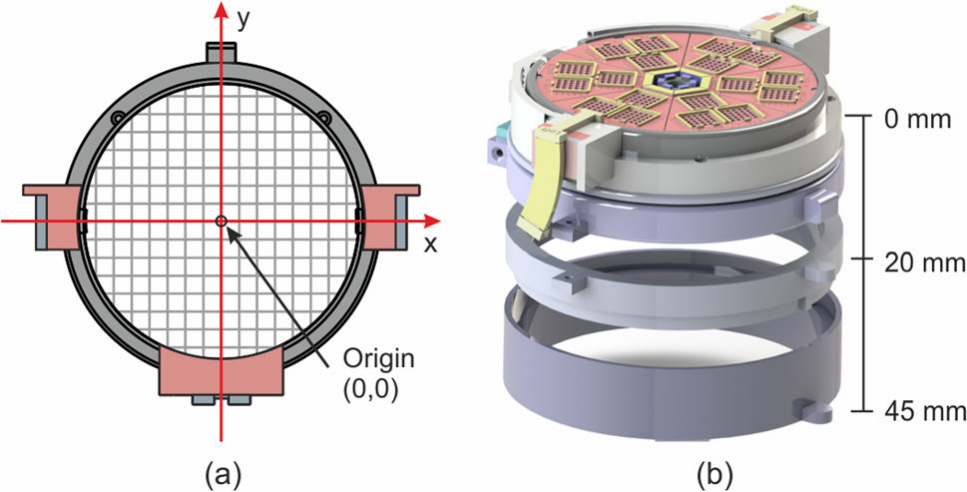
Experiment setup for evaluating robot performance, featuring **a** the origin of the robot coordinate system and **b** varying frame heights to introduce specific depth to the target location

Users operate the robot by inserting the desired angle target along the *z*-axis and *x*-axis for the robot grid, and the required number of steps for the pneumatic motor is calculated based on the motor resolution. No feedback is implemented in the system, thus the relative angle from the previous grid position is employed to monitor the current orientation of the grid system. To ensure proper operation, the robot position must be calibrated initially by positioning it at the home position, a configuration in which no rotation occurs along the *x* and *z* axes. Following this, the registration process is performed to aligns the needle sensor with the robot’s coordinate system. The origin of the robot coordinate system was set at the center of the robot grid (Fig. 4a).

During the test, target points were initially positioned beneath the robot’s body. Later, two different frame heights, 20 and 45 mm, were used to vary the depth of the target locations (Fig. 4b). Additionally, a gelatin-based phantom was placed between the robot and the target points to simulate realistic tissue-needle interactions during insertion.

In summary, the general procedure of the experiment proceeds as follows. First, the user sets the target point and the orientation of the needle. Next, the control algorithm calculates the number of steps required by the pneumatic motor to achieve the correct orientation, as well as the position of the grid hole that reaches the target point. Subsequently, the pneumatic motor move the robot grid along the *x* and *z*-axes to reach the specified orientation. Finally, the user manually inserts the needles through the hole determined by the information obtain from the control algorithm. It is important to note that the needles are not inserted until the robot has completely moved to the correct orientation.

### System accuracy test

The accuracy of the needle placement was evaluated using a series of experiments involving target points positioned at various locations under the robot. These target points comprised 10 randomly selected points within the robot’s reachable regions. The robot’s grid underwent combinations of orientation in the *z* and *x*-axes to simulate different insertion angle. The insertion angle varied from 0 to 60^∘^ in the *z*-axis with 5^∘^ increments and from 0 to 18^∘^ in the *x*-axis with 1.5^∘^ increments, resulting in a total of 13 orientation combinations. Once all points were targeted with every orientation combinations, the robot returned to the home position before repeating the entire process three times. Furthermore, to account for insertion depth, frames and gelatin-based phantoms were incorporated into the test setup. Subsequently, the accuracy test was repeated following the previous procedure.

The selection of the grid hole for insertion relies on information regarding the target point and the current orientation of the robot grid. The nearest grid hole, aligned with the needle orientation to reach the target point, is chosen to minimize targeting error. The reported error in this test represents the average Euclidean distance in the *x* and *y*-axes between the target position and the needle tip position. Notably, errors in the *z*-axis were not considered in this analysis. The robot guides the needle to the desired position, while the actual insertion is manually performed by the user. This mimics the clinical procedure, where clinicians have full control over the insertion process for regulatory and safety reasons. Additionally, the *z*-position of the target points was manually defined by incorporating the frame height into the setup. This could result in *z*-direction accuracy outcomes that may not entirely represent the robot’s performance.

**Fig. 5:**
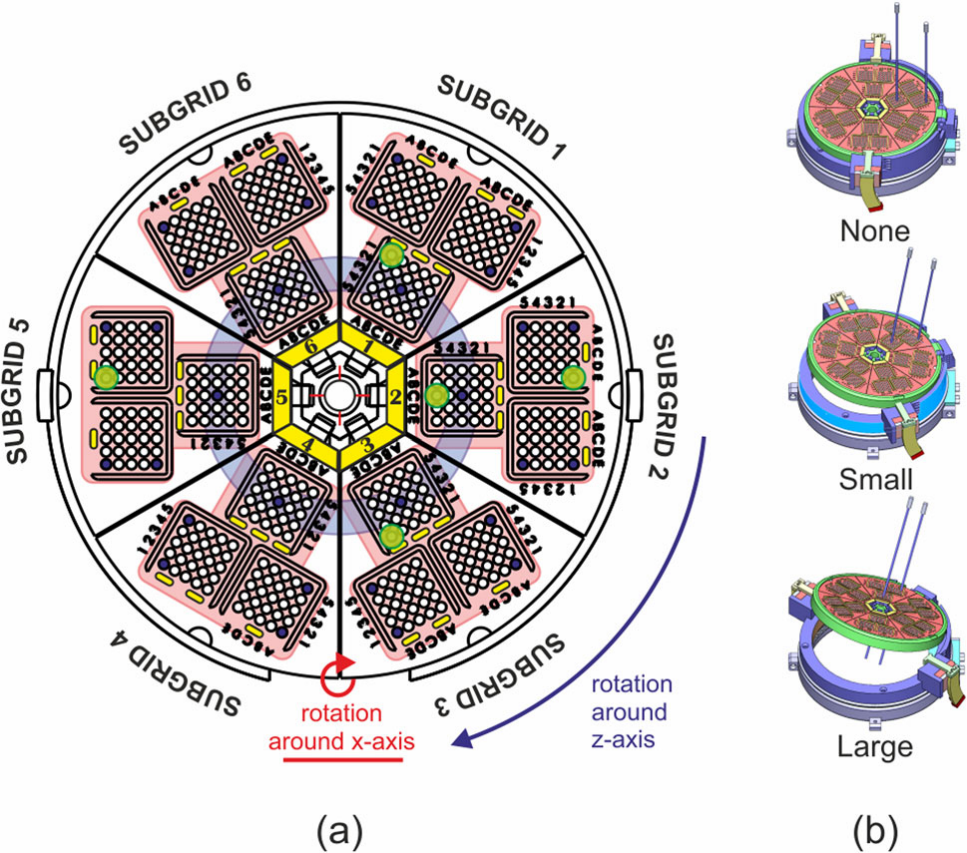
**a** Target holes for needle alignment test: red region for single subgrid test, blue region for all subgrid test, and green region for rigidity test. **b** Various robot configuration in the alignment test: None, Small, and Large

### Needle alignment test

This test evaluated the robot grid’s ability to maintain needle orientation across various robot configurations. Three tests were conducted, as illustrated in Fig. 5a.

In the first test, needle orientation was assessed when inserted through several holes within one subgrid, including ‘x-i-A1’, ‘x-i-A5’, x-ii-E1, x-ii-E5, and x-iii-C5, with x indicated the subgrid number.

The second test compared needle orientations among different subgrids inserted through hole ’x-iii-C5’, also marked by x for the subgrid number. This test aimed to ensure that the robot is able to maintain consistent orientation regardless of the selected insertion hole from any subgrid.

The third test examined the robot’s rigidity in maintaining its position during multiple needle insertions across various grid positions: top (’1-iii-A1’), left (’5-ii-A1’), right (’2-i-E1’), bottom (’3-iii-E1’) and center (’2-iii-C5’). For this test, a needle was first inserted through a selected grid hole, and its orientation recorded. Subsequent needles were then inserted into other grids to simulate multiple insertions. After completion, the orientation of the first needle was recorded again. The comparison between the first needle’s orientation before and after multiple insertions determined the robot’s capability to maintain its grid position in handling multiple needle insertion.

In these three test, the impacts of grid rotation on needle alignment were investigated. Three different positions were evaluated during the recording of needle orientation. These positions included no rotation, small rotation, and large rotation applied in the *x* and *z* axis as shown in Fig. 5b. Additionally, different frame heights were introduced in the third test to account for insertion depth effect on robot rigidity.

Needle orientations were recorded upon reaching the target position, with three repetitions for each hole in each robot configuration.

## Results

### System accuracy test

**Fig. 6:**
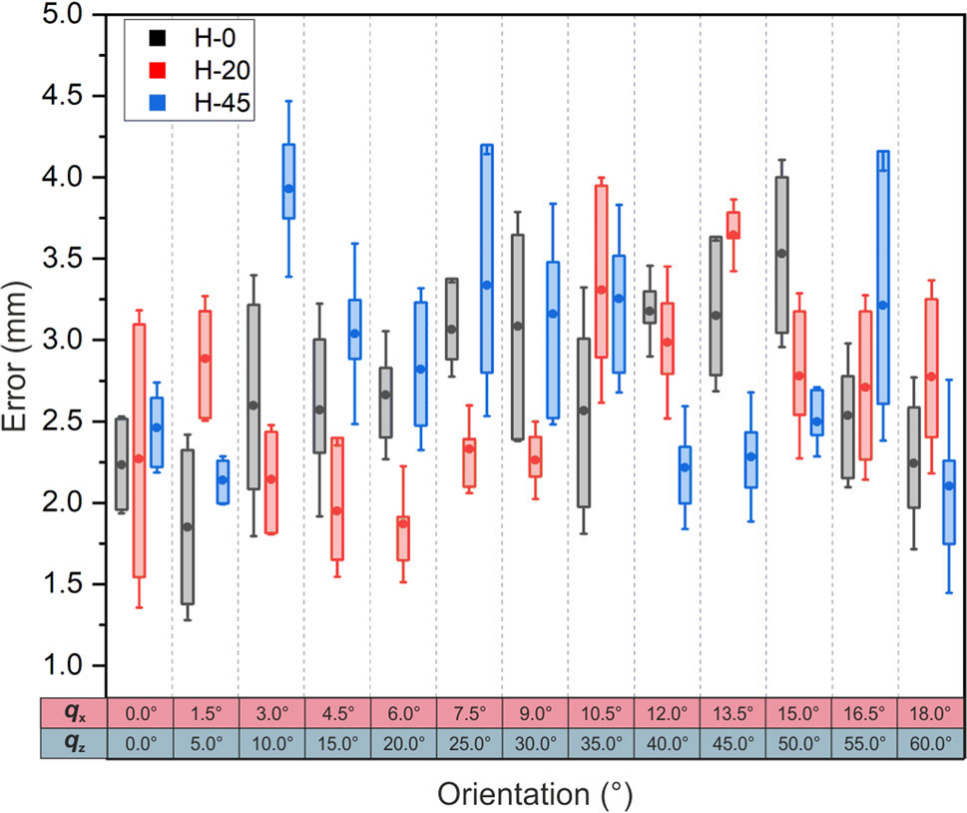
Needle targeting error versus the needle insertion angle and insertion depth. Insertion angle is introduce by applying rotation to the robot in *z* and *x*-axis. Rotation in *z*-axis is increased from 0^∘^ to 60^∘^ in increments of 5^∘^, and rotation in *x*-axis is increased from 0^∘^ to 18^∘^ in increments of 1.5^∘^

For 13 combination of orientation, a comprehensive evaluation to the robot accuracy was conducted by performing 390 insertions, which consist of 3 repetitions for 10 random target points located below the robot base. To minimize the impact of the target hole distribution on the robot’s accuracy, the result from all subgrids were taken into account during the accuracy assessment. The final outcome from the accuracy test is presented in Fig. 6.

When the target plane was directly under the robot body at $H=0$ mm, the mean error across various robot orientations was $2.71\pm 0.65$ mm. Similarly, consistent results were observed when an additional frame was incorporated into the test to introduce depth to the target. Needle insertion through the gelatin phantom at depths of $H=20$ mm and $H=45$ mm yielded average errors of $2.61\pm 0.66$ mm and $2.80\pm 0.72$ mm, respectively.

The ANOVA test revealed that the orientation of the robot body significantly influences accuracy results $(p < 0.0005)$. On the other hand, introducing variations in target depth by adding frames to the robot setup did not yield a statistically significant effect on the accuracy outcomes $(p = 0.14)$.

Overall, these findings highlight the robot’s ability to maintain accuracy across various robot configurations and target depths.

### Needle alignment test

The deviation of needle orientation in individual subgrid was evaluated in the first test. The orientations of the needle was recorded and the average results were calculated for each subgrid. These findings are detailed in Table 1. Generally, needle orientation deviations were below 1 degree for both $R_{x}$ and $R_{z}$ components across all subgrids and grid rotations. However, an exception occurred in subgrid 6 when a large rotation was applied, resulting in the highest deviation of $1.10^{\circ }\pm 0.54^{\circ }$.

Evaluation for orientation deviation for all subgrid was conducted in the second test. Needle orientation deviations were slightly higher compared to the first test. Analysis across all subgrids revealed a maximum deviation of $1.52^{\circ }\pm 0.34^{\circ }$, with deviation exceeding 1^∘^ for all grid orientation, as illustrated in Table 2.

The robot rigidity was checked in the third test. Deviations of the needle before and after multiple insertions are presented in Table 3, with overall deviations below $0.5^{\circ }$. Introducing depth in the rigidity test did not significantly impact needle deviation. However, consistently higher needle deviations were observed when a large orientation was applied to the robot configuration.

**Table 1:** Deviation of the needle orientation in degree (^∘^) for individual subgrid test

Orientation	Ind. grid 1		Ind. grid 2	
	$R_{x}$	$R_{z}$	$R_{x}$	$R_{z}$
None	$0.75\pm 0.33$	$0.59\pm 0.25$	$0.53\pm 0.24$	$0.54\pm 0.25$
Small	$0.55\pm 0.24$	$0.59\pm 0.26$	$0.55\pm 0.30$	$0.60\pm 0.22$
Large	$0.86\pm 0.11$	$0.84\pm 0.35$	$0.66\pm 0.35$	$0.76\pm 0.34$

Orientation	Ind. grid 3		Ind. grid 4	
	$R_{x}$	$R_{z}$	$R_{x}$	$R_{z}$
None	$0.61\pm 0.24$	$0.80\pm 0.26$	$0.53\pm 0.13$	$0.72\pm 0.31$
Small	$0.65\pm 0.19$	$0.58\pm 0.20$	$0.46\pm 0.23$	$0.57\pm 0.27$
Large	$0.72\pm 0.32$	$0.61\pm 0.26$	$0.43\pm 0.19$	$0.52\pm 0.37$

Orientation	Ind. grid 5		Ind. grid 6	
	$R_{x}$	$R_{z}$	$R_{x}$	$R_{z}$
None	$0.57\pm 0.29$	$0.65\pm 0.30$	$0.46\pm 0.18$	$0.58\pm 0.24$
Small	$0.57\pm 0.25$	$0.48\pm 0.21$	$0.57\pm 0.28$	$0.55\pm 0.39$
Large	$0.41\pm 0.28$	$0.75\pm 0.32$	$0.66\pm 0.26$	$1.10\pm 0.54$

**Table 2:** Deviation of the needle orientation in degree (^∘^) for all subgrid test

Orientation	All grid	
	$R_{x}$	$R_{z}$
None	$1.21\pm 0.32$	$1.39\pm 0.30$
Small	$1.52\pm 0.34$	$1.20\pm 0.22$
Large	$1.25\pm 0.26$	$1.37\pm 0.20$

**Table 3:** Deviation of the needle orientation in degree (^∘^) for rigidity test

Orientation	*H*-0		*H*-20		*H*-45	
	$R_{x}$	$R_{z}$	$R_{x}$	$R_{z}$	$R_{x}$	$R_{z}$
None	$0.05\pm 0.05$	$0.09\pm 0.09$	$0.04\pm 0.04$	$0.04\pm 0.04$	$0.05\pm 0.06$	$0.04\pm 0.04$
Small	$0.34\pm 0.78$	$0.27\pm 0.48$	$0.03\pm 0.03$	$0.03\pm 0.02$	$0.07\pm 0.07$	$0.07\pm 0.07$
Large	$0.20\pm 0.17$	$0.36\pm 0.88$	$0.09\pm 0.10$	$0.13\pm 0.11$	$0.14\pm 0.15$	$0.12\pm 0.10$

## Discussion

The study presented the performance of the robot grid system for parallel needle insertion with support to angulate needle insertion path to accommodate targeting the lesion in various position. Two sets of test were conducted to assess the accuracy of the system and its ability to maintain needle orientation.

The system accuracy test result revealed that the robot can uphold needle insertion accuracy across different target depths and orientation. IRE treatment typically utilized to address tumor with size ranging from 1 to 7 cm as outlined by Moir et al. [14]. The robot is expected to position the needle with an error of less than $\pm {5}\,\hbox {mm}$ to adequately cover the minimum tumor size. The accuracy test confirms that the robot meets this requirement, with a maximum error the robot can meet the requirement with average error of $2.71\pm 0.68$ mm.

The result of needle alignment test demonstrated that the proposed robotic grid system effectively maintains parallelism during needle insertion with minimal deviation, even when performed on different subgrids. According to the IRE protocol, a maximum angulation of $10^{\circ }$ is permissible to ensure a homogeneous distribution of the electric field [15]. Additionally, in our previously study [16], it was found that deviation of needle orientation exceeding 5^∘^ can significantly impact the outcome of IRE procedures. In this case, the robot can manage multiple needle insertion with deviation generally below 2^∘^, which is within an acceptable range for IRE procedure.

**Fig. 7:**
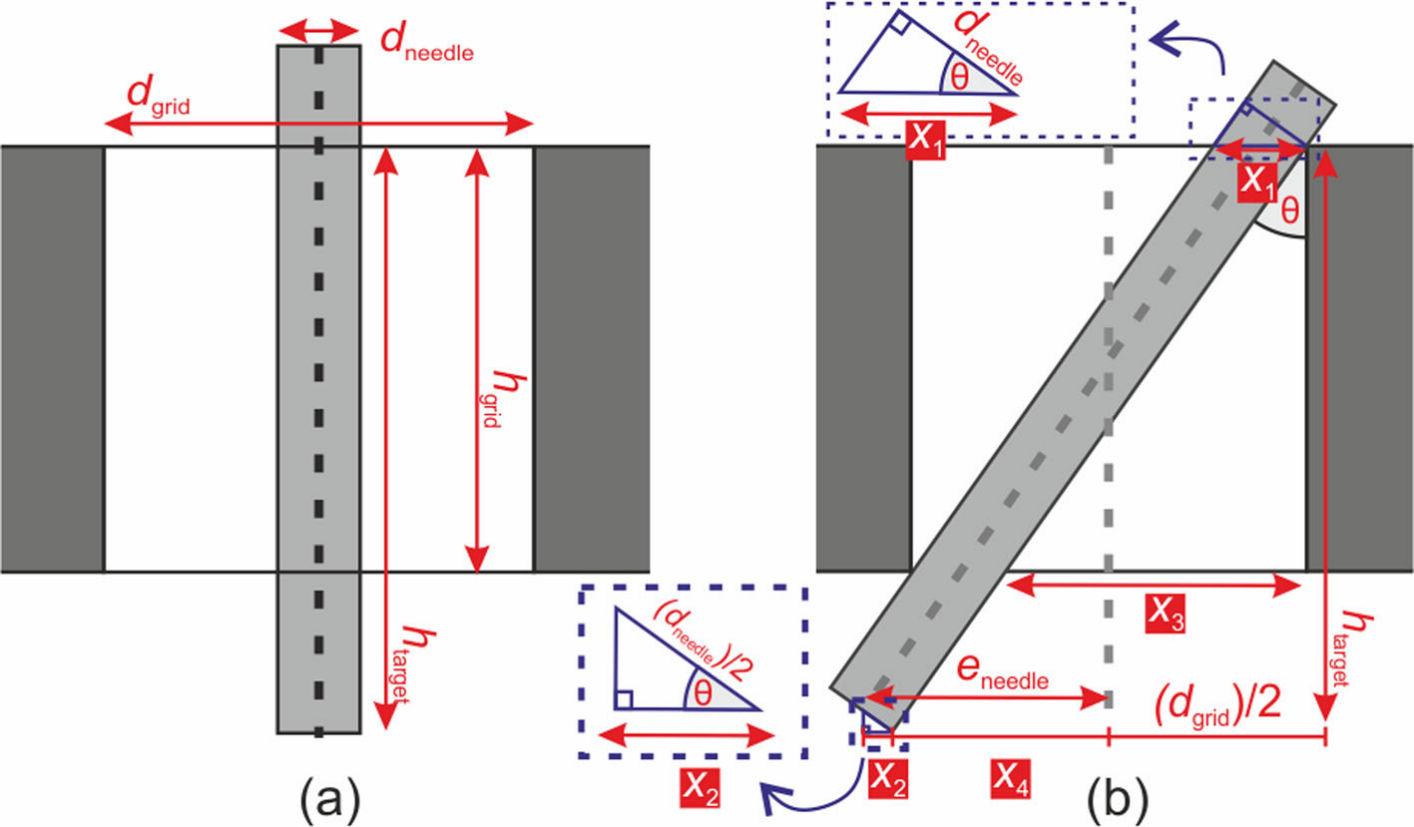
Illustration of needle position inside the hole grid, where **a** needle with a straight trajectory and **b** needle deviation due to the space presence in the hole grid

In an ideal case, performing needle insertion using the robot grid would give precise needle placement. However, the potential sources of error in the experimental procedure may come from a variety factors. It is including but not limited to: inaccuracies in the manufacturing of the robotic components, the rigidity of the robot design, registration errors between the robotic coordinates and the electromagnetic tracker, and potential bending of the needle after repetitive test. Based on our observation during the experiment, it revealed that the another factor of error is coming from the design of the grid hole used in the robot system. To demonstrate this phenomenon, an illustration of the needle positions within the grid hole is shown in Fig. 7.

Ideally, a needle will follow a straight trajectory when inserted through a hole (Fig. 7a). However, due to limitations encountered during manufacturing of the grid components, a certain degree of space may be present within the hole grid, which may cause the needle to deviate from its intended trajectory, as demonstrated in Fig. 7b. In such scenario, the maximum deviation of the needle can be quantified by calculating the values of $x_1$, $x_2$, $x_3$, and $x_4$.

Assuming $\theta$ is small enough, where $\cos \theta \approx 1$, and knowing the dimension of $d_\text {needle}$ and $d_\text {grid}$ as the diameter of the needle and the grid, the value of $x_1$, $x_2$ and $x_3$ are as follows:

2 $$\begin{aligned} x_1&\approx d_\text {needle} \end{aligned}$$

3 $$\begin{aligned} x_2&\approx \frac{d_\text {needle}}{2} \end{aligned}$$

4 $$\begin{aligned} x_3&= d_\text {grid} - x_1 \end{aligned}$$

Furthermore, with $h_\text {grid}$ as the thickness of the grid and $h_\text {target}$ as the depth of the target, $x_4$ can be calculated using the extrapolation made by the needle:

5 $$\begin{aligned} x_4&= \frac{h_\text {target}}{h_\text {grid}}(d_\text {grid}-d_\text {needle}) - \frac{d_\text {grid}}{2} \end{aligned}$$

Finally, needle deviation ($e_\text {needle}$) can be calculated by substituting the value of $x_2$ and $x_4$:

6 $$\begin{aligned} e_\text {needle}&= x_2 + x_4 \nonumber \\&= \left( \frac{h_\text {target}}{h_\text {grid}} -\frac{1}{2}\right) (d_\text {grid} - d_\text {needle}) \end{aligned}$$

In the design of our robot, the grid dimensions, $d_\text {grid}$ and $h_\text {grid}$, are equal to 1.5 mm and 9.5 mm, respectively. An offset of 43.3 mm, which represent the distance from the top surface of the grid to the reference point on the robot’s base, must be taken into account when determining the target depth, $h_\text {target}$. Meanwhile, for the needle dimension, the Aurora 5-DOF needle has a diameter of 1.05 mm. By utilizing these values, we can predict the maximum deviation that may occur to the needle when targeting specific position at various depths.

The impact of needle deviation caused by clearance in the grid hole is clearly visible in the result of needle alignment test. When no rotation is applied to the robot, the deviation across all subgrids is relatively similar. However, when a large orientation is applied, the insertion depth varies between subgrids located near the axis of rotation in the x-axis and those located further away. As a result, subgrids 1 and 6, which are located at the farthest distance from the axis of rotation, have a longer distance to reach the target position, leading to higher deviation in the needle’s orientation compared to other subgrids.

The results of the experiments have indicated a clear need for improvement in the design of the grid system, particularly regarding the clearance of the grid holes. Further studies should concentrate on exploring fabrication techniques that can produce a tighter grid for the robot system, which has been demonstrated to improve accuracy, as reported by McGill et al.[17]. It is also crucial to minimize the distance between the device and the skin surface to decrease deviation error. This is supported by the Eq. 6 that shows that $h_\text {target}$ contributes to needle deviation.

Given that the robot was fabricated using non-metallic, non-magnetic, and non-conductive materials, it is necessary to assess its compatibility within the MRI environment. This can be done by evaluating the robot’s performance under MRI guidance and determining the effect of the robot’s presence on MR image quality. Additionally, incorporating MR-visible markers into the robot’s design is crucial for facilitating the alignment of the device’s coordinate system with the MRI’s coordinate system, thereby improving the accuracy of the registration process.

Another crucial aspect that needs attention is the metric used to evaluate the performance of the robot. The current study focuses on the accuracy and precision of the proposed robot in meeting various requirements reported in the literature. However, for the next clinical study, additional metrics are necessary. These include assessing the duration of installation and conducting experiments, as well as the training time required for the operator. External factor such as patient motion and their impact on the robot accuracy should also be considered. Furthermore, acceptance by the clinical community can be evaluated through questionnaires and interviews to gain insight into translating clinical requirements into robot specifications.

Finally, it is crucial to note that the current device is specifically designed for scenarios requiring multiple needles to be inserted in the same orientation around the target tissue. As such, this study can be extended by developing a path planning algorithm that provides automatic suggestions regarding the number of needles, position of needles, and insertion angle, taking into account the patient anatomy, the size and location of the tumor. However, in cases where individual needle adjustments are required, such as to avoid critical structures, a custom design of the subgrid can be implemented to address such cases. Nevertheless, it is preferable to utilize a more complex system that allows adjustment to each needle configuration, even if it extends the overall procedure time.

## Conclusions

In this study, we presented the design and validation of a robotic grid system, capable of preserving needle parallelism during multiple needle insertions. The robot has 2 DOFs that allow for the adjustment of the grid angulation in the *x* and *z* axes through the use of pneumatic stepper motors. We performed a series of tests, including the system accuracy and needle alignment test, to evaluate the performance of the robot. The results showed that the robotic grid system has limited needle deviation and can be used to place multiple needles in parallel. This robot is suitable for IRE procedures and can be used for wider application of needle insertion procedures, such as microwave and radiofrequency ablation.
